# UIM domain-dependent recruitment of the endocytic adaptor protein Eps15 to ubiquitin-enriched endosomes

**DOI:** 10.1186/1471-2121-15-34

**Published:** 2014-09-27

**Authors:** Azad L Gucwa, Deborah A Brown

**Affiliations:** 1 Department of Biomedical Sciences, Long Island University at Post, Brookville, NY 11548-1300, USA; 2 Department of Biochemistry and Cell Biology, Stony Brook University, Stony Brook, NY 11794-5215, USA

**Keywords:** Endocytosis, Early endosome, Ubiquitin-interacting motif, Receptor down-regulation

## Abstract

**Background:**

Eps15 is an endocytic adaptor protein that stimulates clathrin-mediated endocytosis. Among other interactions, Eps15 binds ubiquitin via UIM domains, recruiting ubiquitinated cargo into clathrin-coated vesicles. In EGF-treated cells, Eps15 also localizes to endosomes. The basis of this localization is not known.

**Results:**

We show that accumulation of ubiquitinated cargo can recruit Eps15 to endosomes via UIM domain interactions. First, treatment of SK-Br-3 breast cancer cells, which overexpress the EGFR family member ErbB2, with geldanamycin to promote receptor ubiquitination and endosomal transport, recruited FLAG-Eps15 to endosomes. Two in-frame ubiquitin constructs, PM-GFP-Ub (retained in endosomes after endocytosis), and GFP-FYVE-UbΔGG (targeted directly to endosomes) also recruited Eps15 to endosomes, as did slowing endosome maturation with constitutively-active Rab5-Q79L. Endosomal recruitment required the UIM domains, but not the N-terminal EH domains or central coiled-coil domains, of Eps15. Silencing of the endosomal Eps15 binding partner Hrs did not affect recruitment of Eps15 to ubiquitin-enriched endosomes. In fact, Hrs silencing itself modestly recruited Eps15 to endosomes, probably by accumulating endogenous ubiquitinated cargo. Eps15 silencing did not affect lysosomal degradation of ubiquitinated ErbB2; however, GFP-FYVE-UbΔGG overexpression inhibited internalization of EGFR and transferrin receptor.

**Conclusions:**

We show for the first time that ubiquitin is sufficient for Eps15 recruitment to endosomes. We speculate that Eps15 recruitment to ubiquitin-rich endosomes may reduce the level of Eps15 at the plasma membrane, slowing endocytosis to allow time for processing of ubiquitinated cargo in endosomes.

## Background

Clathrin-mediated endocytosis is used for selective internalization of specific plasma membrane proteins, in functions that include nutrient uptake and down-regulation of signaling receptors [1-3]. Clathrin provides the structural framework for the coat surrounding internalized vesicles, while a complex array of adaptor proteins are required both for coat formation and for recruiting specific cargo proteins to vesicles [3].

One such adaptor protein, Eps15, plays a key role in clathrin-mediated endocytosis [3,4]. Eps15 is a modular protein. Three N-terminal Eps15 homology (EH) domains bind NPF motifs on a variety of other endocytic adaptor proteins [5]. A central coiled-coil domain mediates Eps15 oligomerization [6] and binding to other proteins including intersectin [7] and the Met receptor [8]. Eps15 contains a domain of DPF repeats that bind AP-2, an abundant adaptor protein that links cargo and clathrin [3,9]. Two ubiquitin interacting motifs (UIM domains) are located near the C-terminus of Eps15 [10].

Eps15 is localized at the rims of clathrin-coated pits [11-13], and is thought to participate in formation of the clathrin lattice. Consistent with this idea, dominant-negative Eps15 constructs inhibit endocytosis of diverse cargoes, including transferrin receptor and the epidermal growth factor receptor (EGFR) [14]. Furthermore, Eps15 stimulates the rate of clathrin coat formation by the clathrin adaptor AP180 [15].

In addition to this general role in clathrin coat assembly, Eps15 has a special relationship with EGFR and other ubiquitinated endocytic cargo. Eps15 was originally identified as an EGFR substrate [16], and is phosphorylated by EGFR on Tyr 850 [17]. Overexpression of an Eps15 mutant lacking this residue blocks endocytosis of the EGFR, but not the transferrin receptor [17]. Although a substantial pool of Eps15 is constitutively present at the plasma membrane, additional Eps15 is recruited there in response to EGFR signaling [18,19]. UIM domain-dependent binding of Eps15 to EGFR and other ubiquitinated proteins at the plasma membrane has been proposed to recruit ubiquitinated proteins to clathrin-coated pits and facilitate their endocytosis [19-23].

Ubiquitin binding by Eps15 may play another role in endocytosis as well. In addition to binding ubiquitin via UIM domains, Eps15 and other endocytic adaptor proteins (Eps15R and epsins) are themselves mono-ubiquitinated [10]. These proteins can bind each other via ubiquitin-UIM interactions and through other motifs [4], suggesting the existence of a UIM-ubiquitin-based protein network at endocytic sites [10]. Formation of this network could enhance endocytosis by increasing the local concentration of these proteins. In yeast, formation of such a network has been proposed to be the main function of ubiquitin-binding interactions of epsins and the Eps15-like protein Ede1 [24].

Eps15 can localize to endosomes as well as the plasma membrane [18,19,25-27]. Eps15 is essentially undetectable in endosomes in resting cells [11,28,29], but is recruited there following EGFR signaling [18,19]. The function of endosomal Eps15 is not clear, and it is not known how EGFR signaling targets the protein there. Endosomal recruitment might require tyrosine phosphorylation, either of Eps15 itself or another EGFR substrate. Alternatively, as activation of EGFR results in ubiquitination and endosomal delivery of the receptor [30], Eps15 might be recruited to endosomes by binding of its UIM domains to ubiquitinated EGFR. In either case, it is not known whether endosomal recruitment of Eps15 requires Hrs. Hrs, together with its binding partner STAM, forms the ESCRT-0 complex that binds ubiquitinated cargo upon its delivery to endosomes, and then passes it on to downstream ESCRT complexes for eventual degradation in lysosomes [30]. Eps15 can bind Hrs [27,31], but the role of this interaction in EGF-dependent endosomal recruitment of Eps15 is not entirely known. Recently, it was found that in *C. elegans*, ESCRT-0 might be recruited to ubiquitinated cargo at the plasma membrane where it directly interacts with the Eps15 homolog EHS-1, suggesting a more complex relationship with the endosomal sorting machinery than previously imagined [32].

A novel Eps15 splice variant named Eps15b was recently discovered [29]. The N-terminal EH domains present in Eps15 are replaced by a short unique sequence in Eps15b, but the two proteins are otherwise identical. Unlike Eps15, which is largely restricted to the plasma membrane in resting cells, Eps15b is constitutively localized to endosomes, apparently in a complex with Hrs [29]. Eps15b promotes EGFR degradation, presumably through ubiquitin-dependent binding to EGFR [29].

In this paper, we tested the role of ubiquitinated cargo in endosomal recruitment of full-length Eps15. We examined three ubiquitinated proteins that can accumulate in endosomes in the absence of EGFR or other tyrosine kinase activity. The first was the EGFR family member ErbB2. Constitutive binding of ErbB2 to the chaperone Hsp90 is required for its stability: ErbB2 is ubiquitinated, internalized by a clathrin-independent pathway [33], and targeted to multi-vesicular bodies (MVBs) independently of signaling activity when cells are treated with the ansamycin antibiotic geldanamycin, which releases Hsp90 from ErbB2 and its other client proteins [34,35]. We also examined two hybrid proteins containing membrane-targeting signals linked to plasmid-encoded ubiquitin [36]. PM-GFP-Ub is initially targeted to the plasma membrane, but accumulates in endosomes following constitutive internalization, while GFP-FYVE-UbΔGG is targeted directly to early endosomes via a PI(3)P-binding FYVE domain [36]. We found that Eps15 was recruited to ubiquitin-rich endosomes in all three cases. Recruitment did not require Hrs, but did require the UIM domains of Eps15. Together, these results suggest that Eps15 can be recruited to ubiquitin-rich endosomes via UIM-domain interactions.

This behavior is similar to that of epsin, another UIM-domain containing endocytic adaptor protein that can be recruited to ubiquitin-rich endosomes [36]. However, epsin is only recruited to endosomes when its normal binding to clathrin is prevented [36]. By contrast, FLAG-Eps15 was recruited to endosomes regardless of expression levels, and without perturbation of other binding interactions, suggesting a physiologically relevant role. Thus, recruitment of Eps15 to ubiquitin-rich endosomes may act as a rheostat, slowing endocytosis and giving the ESCRT machinery time to process ubiquitinated cargo in endosomes.

## Results

### FLAG-Eps15 is recruited to endosomes in GA-treated SK-BR-3 cells

**Figure 1:**
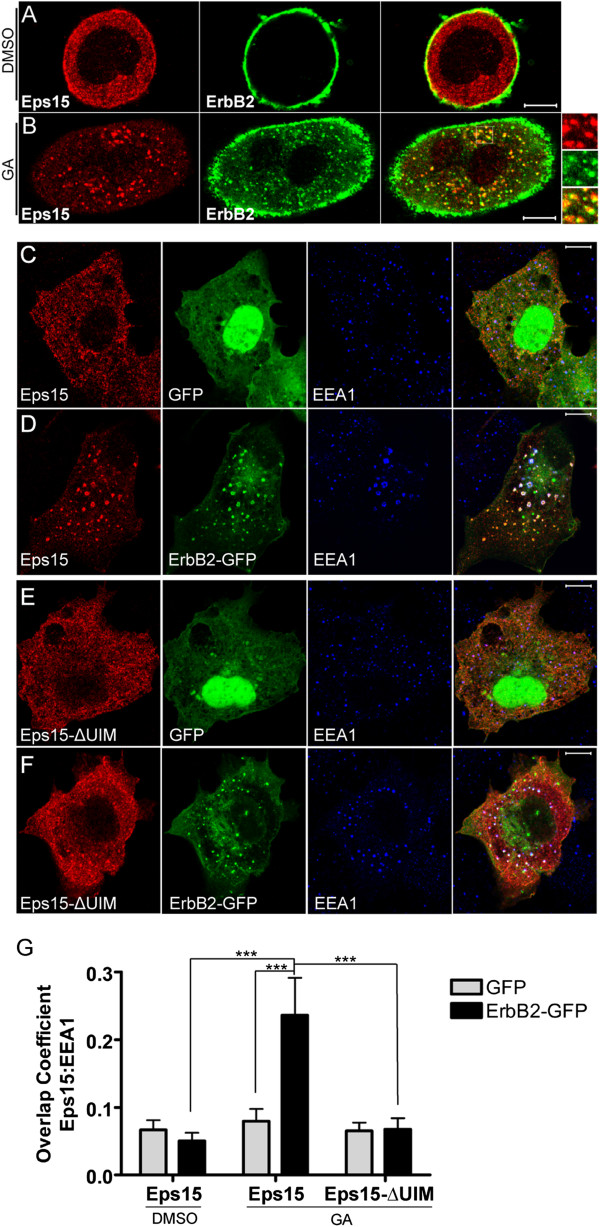
**FLAG-Eps15 is recruited to endosomes enriched in ubiquitinated ErbB2 in a UIM-domain-dependent manner. A**, **B**. FLAG-Eps15-transfected SK-BR-3 cells were treated with DMSO (A) or 5 μM GA (B) for 4 hours, and then processed for IF, detecting FLAG-Eps15 with anti-FLAG antibodies and endogenous ErbB2 with anti-ErbB2 antibodies. **C-F**. COS-7 cells transfected with FLAG-Eps15 or FLAG-Eps15-ΔUIM, together with GFP or ErbB2-GFP as indicated, were treated with 5 μM GA for 4 hours and then processed for IF, detecting FLAG-Eps15 constructs with anti-FLAG antibodies. **G**. Colocalization of FLAG-Eps15 or FLAG-Eps15-ΔUIM with EEA1 in COS-7 cells co-expressing either GFP or GFP-ErbB2 and treated with DMSO or 5 μM GA for 4 hours was quantified using NIH ImageJ. The Manders’ overlap coefficient is shown. Values shown are the average of at least 10 cells in each of 3 experiments, +/- standard error of the mean (SEM). The data were compared using a one-way ANOVA test (***p < 0.0001). Scale bars; 10 μm.

ErbB2 is expressed at high levels on the plasma membrane of SK-BR-3 breast cancer cells (Figure 1A). Transfected FLAG-Eps15 localized to the cytosol and small puncta in these cells, as expected (Figure 1A). GA induces ErbB2 ubiquitination (Additional file 1: Figure S1), internalization, and transport to early and late endosomes and lysosomes, where it can be detected by immunofluorescence (IF) microscopy [33,37]. Endosomal accumulation of ErbB2 is easily detected by immunofluorescence after 4 hours of GA treatment, as a significant proportion of the receptor has either degraded or relocalized from the plasma membrane to endosomes. Upon GA treatment of transfected SK-BR-3 cells, FLAG-Eps15 re-localized to endocytic organelles, where it colocalized with internalized endogenous ErbB2 (Figure 1B).

To study this behavior further, we expressed wild-type or mutant FLAG-Eps15 in COS-7 cells, and examined colocalization with the early endosome marker EEA1 under various conditions, quantitating the colocalization as described in Methods. When expressed alone (not shown) or together with GFP (Figure 1C), FLAG-Eps15 had a fine punctate distribution, as in SK-BR-3 cells, both with (Figure 1C, G) and without (Figure 1G) GA. In agreement with earlier reports [11,28,29], very little FLAG-Eps15 was present on early endosomes (Figure 1C, G). When FLAG-Eps15 was co-expressed with GFP-tagged ErbB2 in these cells, Eps15 localization was similar to that in cells co-expressing GFP, with little endosomal localization (image not shown; quantitation in Figure 1G). However, after GA treatment, both Eps15 and ErbB2-GFP were strongly recruited to endosomes (Figure 1D, G). This required the UIM domains of Eps15, as a construct lacking these domains [38] did not localize to endosomes even when co-expressed with ErbB2-GFP in GA-treated cells (Figure 1, E-G). Together, these results showed that Eps15 was recruited to endosomes when ubiquitinated ErbB2 accumulated there, in a UIM domain-dependent manner.

To determine whether the endosomal recruitment of Eps15 is related to activation of the EGFR signaling pathway, we treated SK-BR-3 cells with either EGF or GA for 0, 4 or 8 hours and looked for an increase in phosphorylated Akt or MAPK (Additional file 2: Figure S2). We found that GA did not affect total MAPK levels. As noted previously [39,40], GA destabilized Akt. However, GA did not stimulate phosphorylation of either Akt or MAPK, suggesting that GA-induced recruitment of Eps15 to endosomes did not require EGFR signaling.

### Eps15 is recruited to endosomes containing PM-GFP-Ub

**Figure 2:**
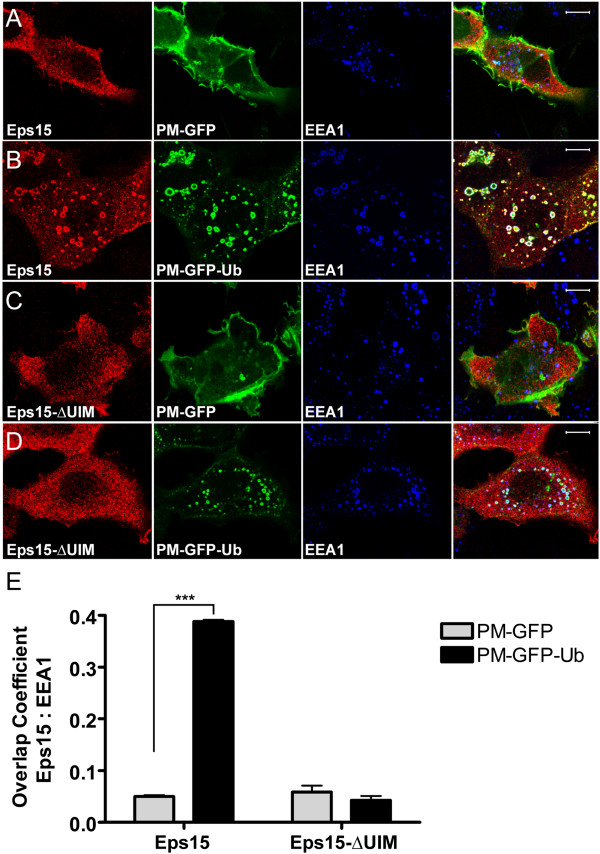
**PM-GFP-Ub recruits FLAG-Eps15 to early endosomes in a UIM-domain-dependent manner. A-D**. COS-7 cells transfected with FLAG-Eps15 or FLAG-Eps15-ΔUIM, together with PM-GFP or PM-GFP-Ub as indicated, were processed for IF, detecting FLAG-Eps15 constructs with anti-FLAG antibodies. **E**. Colocalization of FLAG-Eps15 or FLAG-Eps15-ΔUIM with EEA1 in COS-7 cells co-expressing either PM-GFP or PM-GFP-Ub was determined and is presented as in Figure 1C, averaging values for at least 10 cells in each of 3 experiments, +/- SEM. The data were compared using a one-way ANOVA test (***p < 0.0001). Scale bars; 10 μm.

As GA releases Hsp90 from many proteins [35], it was possible that endosomal recruitment of Eps15 depended on effects that were independent of ErbB2. To provide more direct evidence that ubiquitinated cargo could recruit Eps15 to endosomes, we examined cells expressing PM-GFP-Ub. This construct encodes ubiquitin fused to GFP and to a dually-acylated plasma membrane targeting signal derived from the Src-family kinase Lyn [36,41]. Although PM-GFP-Ub is initially targeted to the plasma membrane, it also accumulates in endosomes, probably by ubiquitin-dependent trapping by the ESCRT machinery following endocytosis ([36] and Figure 2). By contrast, PM-GFP, which lacks plasmid-encoded ubiquitin, is not enriched in endosomes. We found that FLAG-Eps15 accumulated on endosomes in cells expressing PM-GFP-Ub, but not in cells expressing PM-GFP, in a UIM-dependent manner (Figure 2).

### Eps15 is recruited to endosomes containing GFP-FYVE-UbΔGG

**Figure 3:**
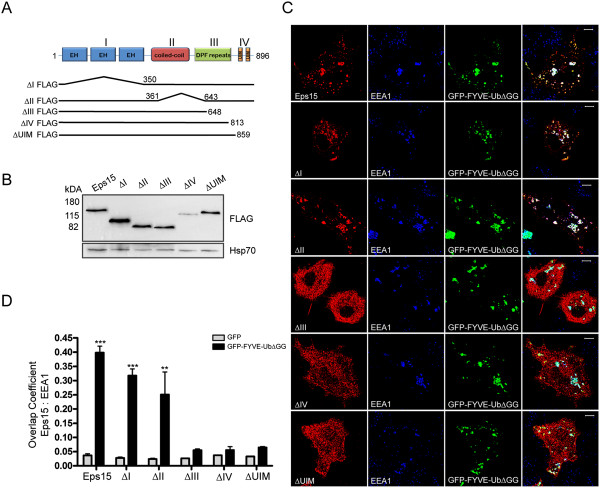
**FLAG-Eps15 recruitment to GFP-FYVE-UbΔGG-enriched endosomes requires UIM, but not EH or coiled-coil domains. A**. Schematic diagram of the Eps15 mutants used. **B**. FLAG-Eps15 (Eps15) or the indicated FLAG-Eps15 mutant was expressed in COS-7 cells. Proteins in cell lysates (equal volumes) were separated by SDS-PAGE and analyzed by Western blotting, probing with anti-FLAG antibodies. The blot was then probed with anti-Hsp70 antibodies to verify equal loading. **C**. Wild-type FLAG-Eps15 (Eps15) or the indicated FLAG-Eps1 mutant was expressed in COS-7 cells together with GFP-FYVE-UbΔGG. Cells were processed for IF, detecting the FLAG-Eps15 proteins with anti-FLAG and then AF-594 goat anti-rabbit antibodies, and EEA1 with anti-EEA1 and then AF-647 goat anti-mouse antibodies. From left to right in each row, anti-FLAG staining, anti-EEA1-staining (pseudo-colored blue), GFP fluorescence, and a merged image are shown. Scale bars; 10 μm. **D**. Wild-type or mutant FLAG-Eps15 was co-expressed with GFP or GFP-FYVE-UbΔGG as indicated, and processed for fluorescence microscopy as in C. Colocalization analysis of the FLAG-Eps15 proteins with EEA1 was performed as in Figure 1. Values shown are the average of 2 experiments, +/- SEM. The data were compared using a one-way ANOVA test (***p < 0.0001, **p < 0.005).

As PM-GFP-Ub is initially targeted to the plasma membrane, it was possible that components required for endosomal recruitment of Eps15 in PM-GFP-Ub-expressing cells – or Eps15 itself - might be transported from the plasma membrane to endosomes together with PM-GFP-Ub. For this reason, we next examined GFP-FYVE-UbΔGG. This construct consists of ubiquitin (lacking the last two residues, in order to prevent linkage of additional ubiquitin moieties) fused to GFP and to the FYVE domain of Hrs, which targets the protein directly from the cytosol to PI(3)P-rich endosomes [36]. When co-expressed in COS-7 cells, FLAG-Eps15 accumulated together with GFP-FYVE-UbΔGG on EEA1-positive endosomes (Figure 3C, top), providing further support for the idea that Eps15 was recruited directly to ubiquitin-rich endosomes.

Surprisingly, some Eps15 was recruited to endosomes in cells expressing GFP-FYVE itself (not shown). We speculate that over-expression of this construct disrupted endosome function by competing with endogenous FYVE domain proteins–including Hrs–for endosome binding. This would cause accumulation of endogenous ubiquitinated cargo in endosomes, recruiting Eps15.

### Neither high FLAG-Eps15 expression nor the FLAG tag is required for Eps15 recruitment to ubiquitin-rich endosomes

Because GFP-FYVE-UbΔGG recruited Eps15 to endosomes efficiently, without possible complications of delivery from the plasma membrane, we used it in further studies to characterize this recruitment in more detail. We could not detect endogenous Eps15 by IF with our antibodies, and we were concerned that endosomal recruitment of FLAG-Eps15 might only occur at high expression levels, after saturation of normal binding interactions. For this reason, we measured endosomal recruitment of FLAG-Eps15 in GFP-FYVE-UbΔGG-expressing COS-7 cells as a function of the level of expression of FLAG-Eps15. Colocalization of FLAG-Eps15 with EEA1 did not correlate with Eps15 expression: FLAG-Eps15 was recruited efficiently to endosomes even in cells with the lowest detectable FLAG-Eps15 level (Additional file 3: Figure S3).

To exclude a possible role for the FLAG tag in endosomal recruitment of Eps15, we showed that mCherry-Eps15 was also recruited to endosomes in cells expressing either PM-GFP-Ub or GFP-FYVEΔGG (Additional file 4: Figure S4).

### The UIM domains of Eps15, but not the EH or coiled-coil domains, are required for recruitment to ubiquitin-containing endosomes

We next mapped the region(s) of Eps15 required for recruitment to ubiquitin-enriched endosomes using a series of FLAG-Eps15 deletion mutants. FLAG-Eps15-∆I lacks the N-terminal EH domains, while FLAG-Eps15-∆II is an internal deletion lacking the central coiled-coil domain ([42]; schematized in Figure 3A). FLAG-Eps15-∆III, FLAG-Eps15-∆IV, and the FLAG-Eps15-∆UIM construct used in Figures 1 and 2 are C-terminal truncation mutants: FLAG-Eps15-∆III lacks most of the DPF-repeat region required for AP-2 binding and the UIM domains, while both FLAG-Eps15-∆IV and FLAG-Eps15-∆UIM lack the UIM domains ([38,42]; Figure 3A). Except for FLAG-Eps15-∆IV, which for unknown reasons was poorly expressed, the constructs were expressed at similar levels in COS-7 cells (Figure 3B).

We next expressed the Eps15 constructs together with GFP or GFP-FYVE-UbΔGG in COS-7 cells and measured colocalization of the Eps15 proteins with EEA1. Selected images are shown in Figure 3C, and quantitation of the colocalization is shown in Figure 3D. FLAG-Eps15-∆I and FLAG-Eps15-∆II were recruited quite efficiently to endosomes in cells expressing GFP-FYVE-UbΔGG, showing that the EH and coiled-coil domains were not essential for endosomal localization (Figure 3C, D). The modest decrease in endosomal recruitment of FLAG-Eps15-∆II, compared to wild-type Eps15, might result from the inability of this construct to oligomerize [43]. As UIM domains bind ubiquitin with low affinity [44], oligomeric Eps15 would be expected to bind more strongly to ubiquitin-rich endosomes than monomeric Eps15, even if binding occurred only through UIM-ubiquitin interactions. By contrast, constructs lacking the UIM domains (FLAG-Eps15-∆III, FLAG-Eps15-∆IV, and FLAG-Eps15-∆UIM) showed very little GFP-FYVE-UbΔGG-dependent endosomal recruitment (Figure 3C, D).

### Tyr 850 is not required for endosomal recruitment of FLAG-Eps15

The EGFR phosphorylates Eps15 on Tyr 850 [17]. To determine whether this modification is required for endosomal recruitment, we co-expressed a non-phosphorylatable Eps15 mutant, Eps15-Y850F, with GFP or GFP-FYVE-UbΔGG in COS-7 cells. In cells expressing GFP, Eps15-Y850F had the same punctate localization as wild-type FLAG-Eps15, and was not present on EEA1-positive endosomes (Additional file 5: Figure S5, top row). However, Eps15-Y850F was efficiently recruited to endosomes in cells expressing GFP-FYVE-UbΔGG (Additional file 5: Figure S5) as well as those containing activated EGFR (Additional file 6: Figure S6, bottom panel). This showed that tyrosine phosphorylation on Tyr 850 is not required for endosomal localization of Eps15.

### Hrs is not required for ubiquitin-dependent recruitment of Eps15 to early endosomes

**Figure 4:**
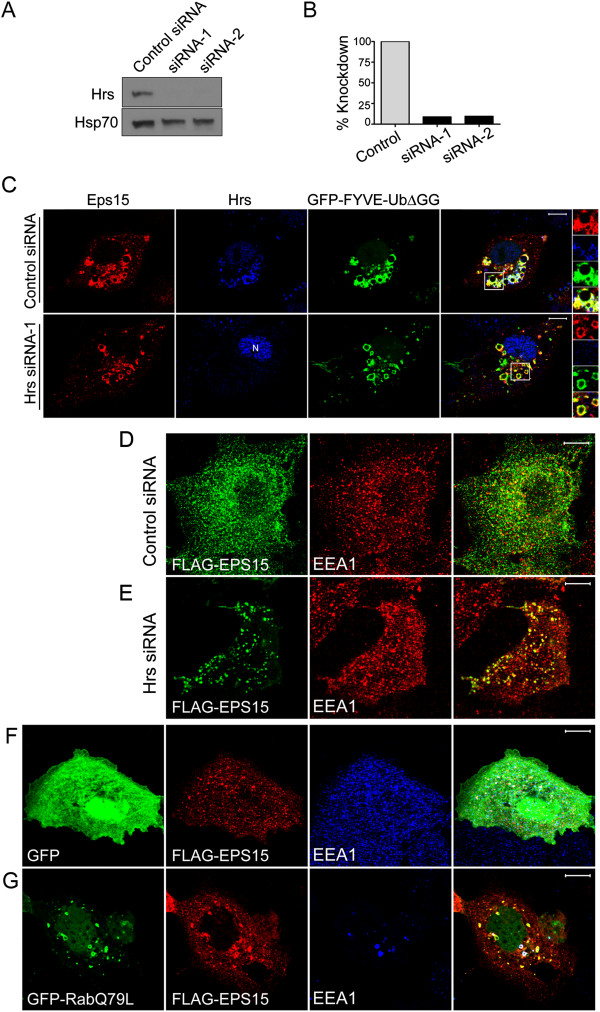
**Hrs is not required for recruitment of Eps15 to GFP-FYVE-UbΔGG or endogenous ubiquitinated cargo.** COS-7 cells were transfected with siRNA targeting Hrs or control siRNA, and also with plasmids encoding FLAG-Eps15 and GFP-FYVE-UbΔGG. **A**. Proteins in lysates (equal volumes from each sample) were separated by SDS-PAGE and then analyzed by Western blotting, probing with anti-Hrs antibodies (top) or anti-Hsp70 antibodies as a loading control (bottom). **B**. Bands on the blot shown in Panel A were quantitated by densitometry, normalizing to the signal in the control sample. **C**. Cells transfected with the indicated constructs were processed for IF microscopy, staining with anti-FLAG and AF-594 goat anti-rabbit IgG to detect FLAG-Eps15 and with anti-Hrs and AF-647 goat anti-mouse IgG to detect Hrs (pseudo-colored blue). **D**, **E**. COS-7 cells transfected with control siRNA (D) or Hrs siRNA-2 (E) and with FLAG-Eps15 were processed for IF microscopy, detecting FLAG-Eps15 with anti-FLAG antibodies and AF-488 goat anti-rabbit IgG, and EEA1 with anti-EEA1 antibodies and AF-594 goat anti-mouse IgG. **F**, **G**. COS-7 cells co-transfected with FLAG-Eps15 together with either GFP or GFP-Rab5Q79L were processed for IF microscopy, detecting FLAG-Eps15 with anti-FLAG antibodies and AF-594 goat anti-rabbit IgG, and EEA1 with anti-EEA1 antibodies and AF-647 goat anti-mouse IgG (pseudo-colored blue). Merged images are shown at the right. Scale bars; 10 μm.

Hrs can bind Eps15 in the cytosol and on endosomes. Eps15b, which localizes primarily to endosomes even without ubiquitin enrichment, binds Hrs *in vivo*[29]. Furthermore, Eps15b localizes to Hrs-positive endosome microdomains, suggesting that Hrs recruits Eps15b to endosomes [29]. Eps15 can also bind Hrs [27,31], though this interaction does not occur at significant levels in cells with normal, low levels of endosomal ubiquitin [29]. For this reason, we next suppressed Hrs expression with siRNA to determine whether Hrs were required for ubiquitin-dependent endosomal recruitment of Eps15. We first verified that Hrs expression was efficiently suppressed (by about 90%) by siRNAs targeting two different sequences in human Hrs (Hrs siRNA-1 and Hrs siRNA-2), both in human HeLa cells (not shown) and in COS-7 cells (Figure 4A, B). We then co-expressed FLAG-Eps15 and GFP-FYVE-UbΔGG, in COS-7 cells previously transfected with either Hrs siRNA-1 or a control siRNA (Figure 4C). In agreement with the Western blot data (Figure 4A, B), Hrs was easily detectable on endosomes in control cells, but not in cells expressing Hrs siRNA-1. (The anti-Hrs antibodies sometimes stained nuclei non-specifically, as seen in Figure 4C). FLAG-Eps15 was recruited efficiently to GFP-FYVE-UbΔGG, PM-GFP-Ub and ErbB2-GFP enriched endosomes even in cells with no detectable Hrs, showing that Hrs was not required for ubiquitin-dependent endosomal recruitment of Eps15 (Figure 4C). Similar results were obtained in cells expressing Hrs siRNA-2 instead of Hrs siRNA-1 (not shown). Furthermore, Hrs silencing did not affect FLAG-Eps15 recruitment to endosomes in COS7 cells expressing PM-GFP-Ub, or in GA-treated SK-BR-3 cells (Additional file 7: Figure S7).

### FLAG-Eps15 is recruited to endosomes enriched in endogenous ubiquitinated cargo

If endosomal recruitment of Eps15 is physiologically relevant, it must occur at reasonable levels of endosomal ubiquitin. Our finding that FLAG-Eps15 was recruited to endosomes in GA-treated SK-BR-3 cells (Figure 1) showed that artificially high, over-expressed levels of ubiquitinated cargo were not required for this recruitment. As another approach to this question, we took advantage of our finding that Hrs was not required for recruitment of FLAG-Eps15 to endosomes in GFP-FYVE-UbΔGG-expressing cells (Figure 4). As Hrs is required for packaging of ubiquitinated proteins into MVBs [30], endogenous ubiquitinated cargo should accumulate on endosome rims upon Hrs silencing. For this reason, we next examined the localization of FLAG-Eps15 in Hrs-silenced COS-7 cells. FLAG-Eps15 was recruited to endosomes in these cells, but not in control cells (Figure 4D, E), showing that endogenous ubiquitinated cargo was sufficient for this recruitment.

In some early experiments, we noticed that GFP-Rab5, expressed to serve as an endosome marker, recruited FLAG-Eps15 to endosomes. This recruitment was even more pronounced when FLAG-Eps15 was co-expressed with the constitutively-active GFP-Rab5Q79L mutant (Figure 4F, G). We do not know the basis of this recruitment. However, Rab5Q79L enhances early endosome function, causing enlargement of endosomes [45], and is likely to delay conversion of early to late endosomes [46]. We speculate that this may delay processing of ubiquitinated cargo in endosomes, allowing it to accumulate to levels high enough to recruit FLAG-Eps15.

### siRNA suppression of Eps15 does not affect ErbB2 degradation

Eps15 might function in endosomes by aiding the ESCRT-0 complex in collecting ubiquitinated cargo for degradation. To test this idea, we examined the effect of Eps15 silencing on degradation of ubiquitinated ErbB2. We chose ErbB2, rather than EGFR, because Eps15 affects EGFR internalization from the plasma membrane [3,4]. Although this effect may be small [29], it makes it harder to determine whether Eps15 also has a second role in EGFR trafficking at endosomes. By contrast, ubiquitinated ErbB2 bypasses the Eps15-dependent step at the plasma membrane, as it is internalized by a clathrin-independent pathway [33]. Following delivery to endosomes, however, ubiquitinated ErbB2, like ubiquitinated EGFR, is efficiently packaged into vesicles inside MVBs [37].

**Figure 5:**
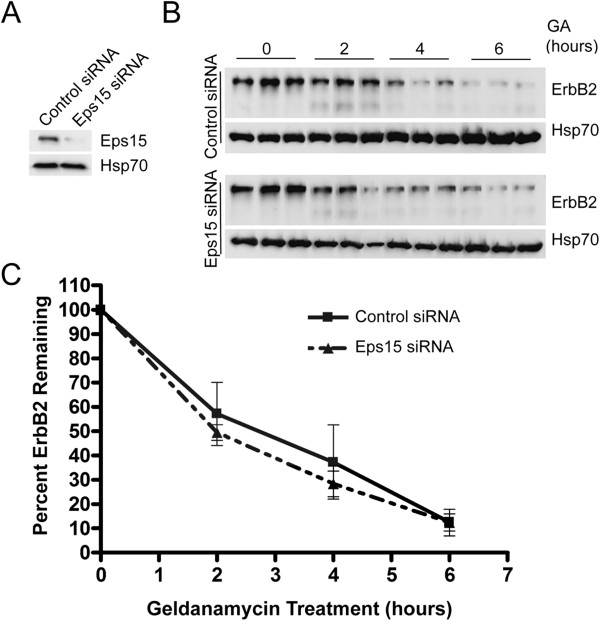
**Eps15 silencing does not affect ErbB2 degradation in GA-treated SK-BR-3 cells. A**. SK-BR-3 cells were transfected with siRNA targeting Eps15 or a control siRNA as indicated. Proteins in equal volumes of cell lysate were separated by SDS-PAGE and analyzed by Western blotting, probing with anti-Eps15 and then anti-Hsp70 antibodies. **B**. Triplicate dishes of SK-BR-3 cells transfected with siRNA targeting Eps15, or a control siRNA, were incubated with 5 μM GA for the indicated times, lysed, and subjected to SDS-PAGE and Western blotting. Equal volumes of each lysate were loaded on the gel. Blots were probed with anti-ErbB2 or anti-Hsp70 antibodies, and then with HRP-conjugated secondary antibodies for detection by chemiluminescence. **C**. SK-BR-3 cells were transfected and processed as in B, except that blots were probed with AF-680-conjugated secondary antibodies for quantitation of bands using the Odyssey infrared imaging system and the associated software. Results shown are the average of 3 separate experiments, +/- SEM.

Roxrud et al. reported that siRNA directed against a sequence present in both Eps15 and Eps15b delayed EGFR degradation in HeLa cells, while an siRNA that targeted a sequence present only in Eps15 had no effect [29]. We tested an siRNA that targeted a sequence present in both Eps15 and Eps15b [29], used previously by others to suppress Eps15 expression [38,47]. This siRNA suppressed expression of full-length (150 kDa) Eps15 by about 80% in SK-BR-3 cells (Figure 5A). We could not reproducibly detect the 120 kDa Eps15b band on Western blots of lysates from a typo for SK-BR-3 or COS-7 cells using our antibodies, so we could not assess the efficacy of Eps15b silencing (not shown). Nevertheless, silencing Eps15 did not affect ErbB2 degradation in GA-treated SK-BR-3 cells (Figure 5B, C) and Eps15 expression was not affected with GA treatment (Additional file 8: Figure S8).

### GFP-FYVE-UbΔGG slows endocytosis of EGFR and transferrin receptor

**Figure 6:**
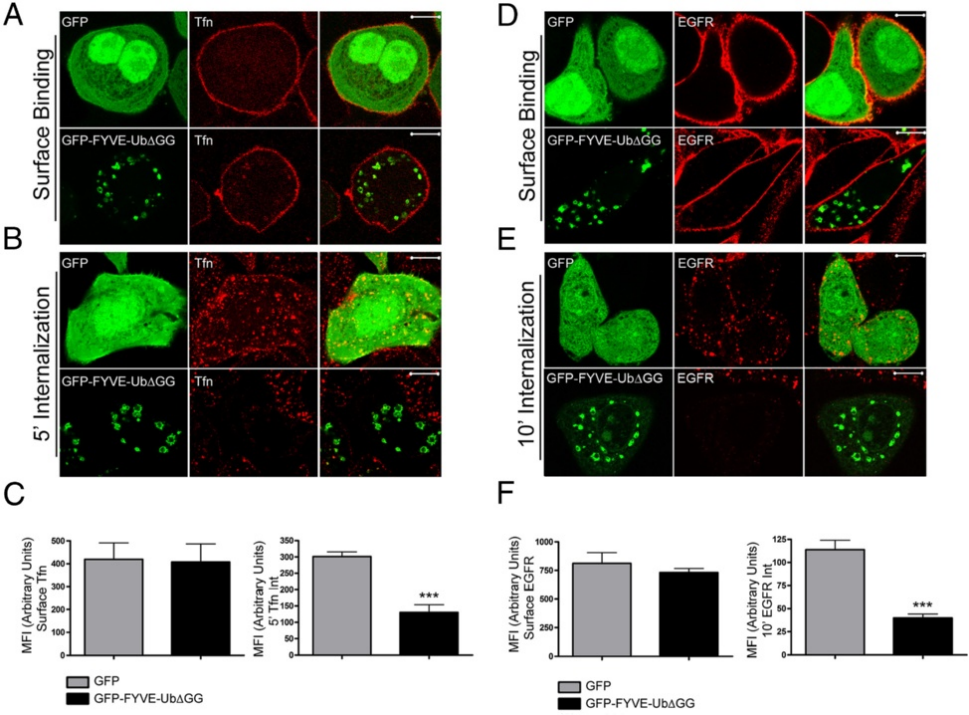
**GFP-FYVE-UbΔGG expression inhibits endocytosis of EGFR and transferrin receptor in SK-BR-3 cells.** GFP or GFP-FYVE-UbΔGG as indicated was expressed in SK-BR-3 cells. A,D. AF-594 transferrin **(A)** or anti-EGFR antibodies **(D)** were bound to cells at 4°C, as detailed in Methods, before fixation. B, E. AF-594 transferrin **(B)** or anti-EGFR antibodies **(E)** were allowed to internalize at 37°C as detailed in Methods. A,B,D,E; Cells were processed for fluorescence microscopy as in Figure 1, except that cells shown in A, B, and D were not permeabilized. Tfn, transferrin. Scale bars; 10 μm. D, E. Anti-EGFR antibodies were detected with AF-594-goat anti-mouse IgG. C, F. AF-594-transferrin **(C)** or AF-594 goat anti-mouse IgG **(F)** fluorescence was quantitated as described in Methods. In each panel, MFI of cells with surface-bound probes is shown on the left; MFI of cells containing internalized fluorophores is on the right. Values for GFP-expressing cells are shown with gray bars; values for GFP-FYVE-UbΔGG-expressing cells are shown with black bars. Results shown are the average of 2 separate experiments, +/- SEM. The data were compared using a one-way ANOVA test (***p < 0.0001).

Our current working model is that Eps15 recruitment to ubiquitin-rich endosomes may function to sequester the protein away from the plasma membrane. This might provide a feedback mechanism, slowing clathrin-mediated endocytosis when high amounts of ubiquitinated cargo were delivered to endosomes. This could provide time for the ESCRT machinery to clear the ubiquitinated proteins, preventing excessive over-accumulation in endosomes. As a preliminary test of this idea, we measured internalization of endogenous EGFR and transferrin receptor in SK-BR-3 cells expressing GFP-FYVE-UbΔGG. We first found that steady-state levels of both receptors at the plasma membrane were similar in GFP-FYVE-UbΔGG-expressing and control cells (Figure 6A, D). However, GFP-FYVE-UbΔGG expression slowed internalization of both AF-594-transferrin (Figure 6B) and anti-EGFR antibodies (Figure 6E). Quantitation is shown in Figure 6C and F. Interestingly, ubiquitin-dependent recruitment of Eps15 to endosomes affected the rate of transferrin receptor and EGFR endocytosis, but not cell surface levels. It is possible that receptor recycling might partially compensate for an altered internalization rate. It is also possible that we could not measure a small increase in plasma membrane levels of these receptors. Because of membrane infoldings and uneven morphology, quantitation of plasma membrane fluorescence may be slightly less accurate than that of internalized material.

This result is consistent with our model that ubiquitin-dependent recruitment of Eps15 to endosomes might slow endocytosis by reducing the amount of the protein at the plasma membrane. However, much further work will be required to fully support this idea. It is important to note that GFP-FYVE-UbΔGG overexpression might have other unanticipated effects on endosomal dynamics, possibly by interfering with normal endosome funtion, that could contribute to the observed inhibition of endocytosis secondarily. Furthermore, Eps15 may not be the only clathrin coat accessory protein whose recruitment to endosomes might contribute to inhibition of endocytosis. Endosomal ubiquitin could also recruit other ubiquitin-binding accessory proteins, further inhibiting endocytosis.

## Discussion

It is not known how Eps15 is recruited to endosomes in response to EGFR signaling [18,19]. As EGF stimulates tyrosine phosphorylation of the receptor itself, of Eps15, and of other substrates, and also leads to EGFR ubiquitination, any of these events might recruit Eps15 to endosomes. It is also not known whether the endosomal Eps15 binding partner Hrs is required for this recruitment.

In this paper, we showed that Eps15 was recruited to endosomes enriched in ubiquitinated cargo. Recruitment did not require Eps15 Tyr850, which is phosphorylated by EGFR, or Hrs, but required the UIM domains of Eps15. Furthermore, Eps15 showed UIM-domain-dependent recruitment to novel ubiquitin-rich plasma membrane clusters. Together, these findings suggest that binding of Eps15’s UIM domains to ubiquitin can determine its localization in the cell, and that EGF treatment can recruit Eps15 to endosomes by causing ubiquitinated EGFR to accumulate there.

Unlike Eps15, Eps15b is constitutively present on endosomes [29]. Eps15b binds Hrs and colocalizes with Hrs in microdomains on endosomes. Roxrud et al. suggested that while EH domain interactions and AP-2 binding target Eps15 to the plasma membrane, Eps15b is targeted to endosomes by binding to Hrs [29]. In contrast, Mayers et al. recently found that binding of the *C. elegans* homolog of human Eps15, EHS-1, bound to Hrs via the EH domains located on the amino terminus of the protein [32]. Taken together, this suggests the UIM-dependent, Hrs-independent targeting of Eps15 to ubiquitin-rich endosomes that we observed occurs by a different mechanism than constitutive, Hrs-dependent endosomal targeting of Eps15b.

Our findings on ubiquitin-dependent Eps15 targeting are very similar to behavior of epsin reported previously by Chen and DeCamilli [36]. As we found for Eps15, epsin could be recruited to endosomes or other cellular sites in response to ubiquitin accumulation, in a UIM-dependent manner. However, behavior of the two proteins differed in one key way. Epsin, unlike Eps15, binds directly to clathrin [3]. Epsin was only recruited to ubiquitin-enriched endosomes when clathrin binding was prevented, either by mutation of epsin or silencing of clathrin [36]. By contrast, we found that intact Eps15 was readily recruited to ubiquitin-rich endosomes. Both Eps15 and epsin have multiple binding partners at the plasma membrane, and these interactions probably counteract UIM-dependent targeting to endosomes, as shown for clathrin binding by epsin [36]. Eps15 localization is also probably determined by the balance of affinities for its various binding partners. However, our results suggest that UIM domain interactions are more likely to prevail in determining localization of Eps15 than of epsin. Although the affinity of individual UIM domains for ubiquitin is low [44], Eps15 can form dimers and tetramers via its coiled-coil domain [43]. Thus, increasing the local concentration of ubiquitin should greatly increase the avidity of Eps15 oligomers for ubiquitin-rich sites. Our results suggest that this is enough to recruit Eps15 to ubiquitin-rich endosomes. Thus, ubiquitin-dependent targeting appears to occur more easily for Eps15 than for epsin, and may be more likely to play an important physiological role.

In this context, a significant question is whether the ubiquitin-dependent recruitment we observed occurs at physiological levels of Eps15. This is an especially important concern because all our experiments were done using over-expressed Eps15 constructs. It is possible that overexpressed Eps15 might saturate its normal plasma membrane binding partners, artificially creating a pool available for recruitment to endosomes. For this reason, we carefully examined endosomal recruitment in cells expressing the lowest detectable level of FLAG-Eps15 (Additional file 2: Figure S2). We saw the same recruitment of FLAG-Eps15 to ubiquitin-rich endosomes at all levels of FLAG-Eps15 expression, suggesting that endosomal recruitment is not an artifact of overexpression.

Several functions for endosomal recruitment of Eps15 can be imagined. One obvious possibility is to aid the ESCRT-0 complex in processing ubiquitinated cargo for degradation. This could occur by direct binding of Eps15 to ubiquitinated cargo, and/or by establishment of a ubiquitin-dependent protein network analogous to that at the plasma membrane [10,24]. Our finding that Eps15 silencing did not affect ErbB2 degradation (Figure 5) argues against this possibility, and suggests that Eps15 is not uniquely required for degradation of ubiquitinated cargo. However, our findings do not exclude this possibility. Eps15 overlaps functionally with the related adaptor Eps15R and epsin at the plasma membrane [38]. Eps15R, and possibly even epsin, might be recruited to endosomes to substitute for Eps15 after its silencing.

Roxrud et al. found that silencing of Eps15 with an siRNA that spared Eps15b did not affect EGFR degradation. By contrast, an siRNA targeting a sequence present in both Eps15 and Eps15b delayed EGFR degradation [29]. These results suggested that Eps15b, but not Eps15, participates in EGFR degradation. The siRNA that we used was directed against a sequence present in both Eps15 and Eps15b. Thus, our results appear to contrast with those of Roxrud et al. [29]. However, we could not reproducibly detect Eps15b on Western blots, probably because of the cell types and/or anti-Eps15 antibodies we used. Thus, we could not tell how well our siRNA suppressed Eps15b expression. Roxrud et al. reported more efficient suppression of Eps15 than Eps15b, despite using siRNA that targeting a shared sequence [29]. If the same were true for the siRNA we used, sufficient Eps15b might have remained to aid in ErbB2 degradation.

## Conclusions

Here, we show for the first time that ubiquitin alone is sufficient for Eps15 recruitment to endosomes. Although the function of this targeting remains unclear, our findings suggest the following possibility. Extensive delivery of ubiquitinated cargo to endosomes might saturate the ESCRT machinery, causing accumulation of ubiquitinated proteins on the limiting membrane. Sequestration of Eps15 away from its normal site of action at nascent clathrin-coated pits might slow endocytosis, providing time for the ESCRT complexes to catch up and package cargo into the MVB interior in an orderly fashion.

## Methods

### Cells and transfection

COS-7 and SK-BR-3 cells were from American Type Culture Collection (ATCC, Manassas, VA) and were cultured in Dulbecco’s modified Eagle’s medium (DMEM; Invitrogen, Carlsbad, CA) with 10% iron-supplemented calf serum (JRH, Lenexa, KS) and penicillin/streptomycin in a humidified incubator with 5% CO_2_ at 37°C.

SK-BR-3 cells were transiently transfected with Lipofectamine 2000 (Invitrogen) in Optimem I reduced-serum medium (Invitrogen) according to the manufacturer’s recommendation. COS-7 cells were transfected with either Lipofectamine 2000 or 50 kDa poly (2-ethy)-2 oxazoline (polyethylenimine; PEI; Sigma Aldrich, St. Louis, MO), deacylated according to [48]. For PEI transfection, 1 μg DNA and 6 μL 25 mM PEI were added to 0.1 ml of 0.15 M NaCl with vortexing, incubated at room temperature for 10 min, and then added drop wise to cells in normal growth media. Cells were then incubated for 24 hours at 37°C before use.

### Antibodies, fluorescent compounds, and other reagents

Mouse monoclonal anti-ErbB2 N28 used for cell-surface detection in IF experiments was from LabVision (Fremont, CA). Lab Vision mouse monoclonal anti-ErbB2 (Ab-20 cocktail) was used for Western blotting. Anti-FLAG antibodies: for IF, rabbit polyclonal anti-DYKDDDDK was from Pierce Antibodies, Thermoscientific (Rockford, IL). For Western blotting, mouse monoclonal anti-FLAG® M2 antibody and rabbit polyclonal anti-GAPDH was from Sigma-Aldrich. Mouse monoclonal anti-EEA1 and anti-Eps15 (clone 17) were from BD Biosciences (San Jose, CA). Mouse monoclonal anti-EGFR mouse (Ab-12 cocktail) was from Thermoscientific. Rabbit monoclonal anti-EGFR (D38B1), anti-Akt (C67E7), anti-p-Akt (D9E), anti-MAPK (137 F5), anti-p-MAPK (D13.14.4E) were from Cell Signaling Technology (Danvers, MA). Anti-Hrs (A-5) mouse monoclonal was from Alexis Biochemicals, Enzo Life Sciences International, Inc. (Plymouth Meeting, PA). Mouse monoclonal anti-Hsp70 was from Santa Cruz Biotechnology, Inc. (Santa Cruz, CA). Mouse monoclonal anti-Histone H3 (ab1791( was from Abcam (Cambridge, MA). HRP**-**goat anti-mouse IgG and HRP-donkey anti-rabbit IgG were from Jackson Immunoresearch (West Grove, PA). AlexaFluor (AF)-488, AF-594, AF-647 and AF-680-conjugated goat anti-mouse and goat anti-rabbit IgG and AF-594-transferrin were from Invitrogen (Carlsbad, CA). Other reagents: GA (used at 5 μM) was from the Drug Synthesis and Chemistry Branch, National Cancer Institute (Bethesda, MD). EGF was from Calbiochem, EMD Biosciences.

### Plasmids

pEGFP-N1 was from Clontech (Mountain View, CA). Plasmids encoding FLAG-Eps15 and FLAG-Eps15-∆UIM [38], PM-GFP, PM-GFP-Ub and pEGFP-2xFYVE-Ub∆GG [36] were gifts of P. DeCamilli (Yale University, New Haven, CT). pcDNA3 ErbB2-GFP [49] was the gift of P. Liu (Univ. North Carolina, Chapel Hill, NC). Plasmids encoding FLAG-Eps15-∆I, FLAG-Eps15-∆II, FLAG-Eps15-∆III, FLAG-Eps15-∆IV and FLAG-Eps15-Y850F [42] were the gifts of E. Fon (McGill University, Montréal, Canada). A constitutively-active GFP-Rab5-Q79L plasmid [50] was the gift of A. Levey (Emory University, Atlanta GA).

### Fluorescence microscopy

Cells were seeded on acid-washed glass coverslips in 35 mm dishes, transfected, and examined 1 day after transfection. Cells were fixed in phosphate-buffered saline (PBS; 150 mM NaCl, 20 mM phosphate buffer, pH 7.4) containing 3% paraformaldehyde for 30 minutes, and permeabilized at room temperature with PBS containing 0.5% Triton unless otherwise noted. The cells were then blocked with Blocking Buffer (PBS containing 3% BSA and 10 mM Gly). Primary and secondary antibodies were diluted in Blocking Buffer. Cells were incubated with primary antibodies for 1 hour at room temperature, followed by secondary antibodies for 30 minutes, also at room temperature. Cells were photographed and images were captured using a Zeiss inverted Axiovert 200 M microscope with a two-photon laser scanning confocal system. All images were acquired with a 100× oil immersion objective (N.A. = 4.5) and processed with Zeiss LSM software. When necessary, images were further processed using Adobe Photoshop, adjusting contrast and/or brightness for optimal viewing. Each channel was adjusted separately and changes were applied to the whole field.

### Colocalization analysis

Colocalization analysis of confocal images was performed with NIH Image J (http://rsb.info.nih.gov/ij/) and the JaCoP plug-in [51] as described previously [33]. Manders’ overlap coefficients were reported and can be interpreted as percent colocalization. The statistical analysis of this data was performed using Graphpad.

### Western blotting

Proteins were separated by sodium dodecyl sulfate polyacrylamide gel electrophoresis (SDS‐PAGE), and then transferred to nitrocellulose for Western blotting and detection on film by enhanced chemiluminescence as described [52]. Where indicated, bands were scanned and quantified using NIH ImageJ. For quantitating ErbB2 degradation, bands were labeled with AF-680-secondary antibodies and detected and quantified with the Odyssey-Infrared Imaging System (LI-COR Biosciences, Lincoln, NE), using the Odyssey imaging software.

### Immunoprecipitation of ErbB2

SK-BR-3 cells were treated with 5 μM for 1 hour prior to the addition of lysis buffer (1% Triton-X-100, 0.15 M NaCl, 2 mM EDTA, 50 mM NaF plus protease inhibitors). Cells were lysed on ice for 20 minutes, scraped and then spun down for at 4°C for 15 minutes. Rabbit anti-mouse coated protein A beads were added to the lysate for 1 hour at 4°C. Samples were washed twice with PBS, then one time with 25 mM Tris Cl, pH 7.4 before adding Laemmli buffer, followed by Western blotting.

### siRNA silencing

siRNA duplex constructs targeting Hrs and Eps15 were made using Stealth RNAi™ siRNA technology (Invitrogen). Eps15 was targeted using a sequence (AAACGGAGCUACAGAUUAU) near the 3’ end of the coding sequence, as previously described [38,47]. The targeted sequences for Hrs were GCACGGUAUCUCAACCGGAACUACU (siRNA-1), and CAGAAUCUCAUGACCACCCUCCCAA (siRNA-2). The Medium GC StealthRNAi™ siRNA Duplex (Invitrogen) was used as a control. For Hrs knockdown, COS-7 cells were first transfected with FLAG-Eps15 and GFP-FYVE-Ub∆GG using Lipofectamine 2000. After 5 hours, 50 nmole siRNA mixed with additional Lipofectamine 2000 was added. After 24 hours, cells were trypsinized. Half of the cells were replated in a 35 mm dish with a coverslip (for IF analysis); the other half were plated in a separate 35 mm dish for Western blotting to confirm Hrs knockdown. Cells were incubated for a further 24 hours before harvesting. For Eps15 knockdown, SK-BR-3 cells were seeded in 35 mm dishes and transfected using Lipofectamine 2000 and 100 nmole siRNA oligos in Optimem for 24 hours. After 24 hours, Optimem was replaced with DMEM containing 10% calf serum. Cells were incubated an additional 48 hours before assaying for ErbB2 degradation.

### Transferrin binding and internalization

To measure cell-surface transferrin receptor levels, SK-BR-3 cells (transfected as described in the legend to Figure 6) were incubated on ice for 2 hours with 50 μg/ml AF-594-transferrin in growth media, and then processed for IF as described above. To measure transferrin internalization, pre-warmed media containing 50 ng/ml transferrin was added to cells at 37°C for 5 minutes before fixation and processing for IF. Values shown are averages from two separate experiments, analyzing at least 50 cells on each slide. All images were captured using the confocal microscope described above. The same acquisition parameters were used for all images to be compared quantitatively, and images were not further processed after acquisition. Mean intensity fluorescence (MFI) of individual cells was calculated using the histogram macro on the Zeiss LSM software.

### EGFR surface expression and internalization assays

Anti-EGFR antibodies (2 μg/ml) were bound to transfected SK-BR-3 cells for 1 hour at 4°C. To quantitate cell-surface EGFR, cells were fixed but not permeabilized before incubation with AF-594 secondary antibodies and processing for IF as described above. To measure EGFR internalization, antibody-bound cells were incubated with pre-warmed growth media containing 100 ng/ml EGF for 10 minutes at 37°C. Residual surface-bound antibodies were then stripped from cells on ice with acid (100 mM Gly, 50 mM KCl, 20 mM MgOAc, pH 2.3), using 3 washes of 3 minutes each. Cells were then fixed, permeabilized, and incubated with AF-594 goat anti-mouse IgG antibodies. The experiment was repeated 3 times, quantitating MFI of at least 10 cells per slide as described above for transferrin experiments. Background fluorescence, determined as the average MFI in the nuclei of 5 cells, was subtracted from all values for internalized EGFR.

## Abbreviations

AF: AlexaFluor; DMEM: Dulbecco’s modified Eagle’s medium; EGFR: Epidermal growth factor receptor; EH: Eps15 homology; IF: Immunofluorescence; MFI: Mean fluorescence intensity; MVBs: Multi-vesicular bodies; PBS: Phosphate-buffered saline; PEI: 50 kDa poly (2-ethy)-2 oxazoline; SDS-PAGE: Sodium dodecyl sulfate polyacrylamide gel electrophoresis; SEM: Standard error of the mean; Tfn: Transferrin; UIM: Ubiquitin interacting motifs.

## Authors’ contributions

ALG carried out the experiments in this study. DAB conceived of the study, participated in the design of the study and drafted the manuscript. Both authors read and approved the final manuscript.

## Supplementary Material

Additional file 1: Figure S1 ErbB2 is ubiquitinated following GA treatment. SK-BR-3 cells were left untreated or incubated with 5 μM GA for 1 hour, lysed, and immunoprecipitated with anti-ErbB2. Lysates were then subjected to SDS-PAGE and Western blotting. Blots were probed with anti-ErbB2 (top panel) and anti-ubiquitin (bottom panel), and then with HRP-conjugated secondary antibodies for detection by chemiluminescence. Click here for file

Additional file 2: Figure S2 GA does not activate Akt or MAPK. SK-BR-3 cells were either serum starved and treated with 100 ng/ml EGF for 45’ at 37°C, or treated with 5 μM GA for the indicated times, lysed, and subjected to SDS-PAGE and Western blotting. Equal volumes of each lysate were loaded on the gel. Blots were probed with anti-Akt, anti-p-Akt, anti-MAPK, anti-p-MAPK and anti-Histone H3 antibodies (loading control), and then with HRP-conjugated secondary antibodies for detection by chemiluminescence. Click here for file

Additional file 3: Figure S3 Endosomal recruitment of FLAG-Eps15 does not depend on FLAG-Eps15 expression level. FLAG-Eps15 and EEA1 were detected in FLAG-Eps15- and GFP-FYVE-UbΔGG-transfected COS-7 cells with rabbit anti-FLAG and mouse anti-EEA1 antibodies and appropriate secondary antibodies (AF-594 goat anti-rabbit IgG and AF-647 goat anti-mouse IgG). The Manders’ overlap coefficient for colocalization of Eps15 with EEA1 in each of 27 cells is plotted as function of the mean AF-594 fluorescence intensity in the same cell. Click here for file

Additional file 4: Figure S4 Cherry Eps15 is recruited to PM-GFP-Ub and GFP-FYVE-UbΔGG. mCherry-Eps15 was co-expressed in COS-7 cells with GFP (A), PM-GFP-Ub (B) or GFP-FYVE-UbΔGG (C), and cells were processed for IF microscopy. Scale bars; 10 μm. Click here for file

Additional file 5: Figure S5 Tyr 850 is not required for endosomal recruitment of FLAG-Eps15. FLAG-Eps15 Y850F was co-expressed in COS-7 cells with either GFP (top) or GFP-FYVE-UbΔGG (bottom), and cells were processed for IF microscopy. FLAG-Eps15 Y850F was detected with anti-FLAG antibodies and AF-594 goat anti-rabbit antibodies, while EEA1 was detected with anti-EEA1 antibodies and AF-647 goat anti-mouse antibodies (pseudo-colored blue). Click here for file

Additional file 6: Figure S6 Eps15 and Eps15 Y850F are recruited to activated EGFR. FLAG-Eps15 and FLAG-Eps15 Y850F were expressed in SK-BR-3 cells and were either left untreated, or stimulated with 100 ng/ml EGF for 10’ at 37°C and then processed for IF microscopy. FLAG-Eps15 and FLAG-Eps15 Y850F were detected with anti-FLAG antibodies and AF-594 goat anti-mouse antibodies, while endogenous EGFR with anti-EGFR antibodies and AF-488 goat anti-rabbit antibodies. Merged images are shown at the right with DAPI staining. Scale bars; 10 μm. Click here for file

Additional file 7: Figure S7 Hrs is not required for recruitment of Eps15 to PM-GFP-Ub or GA-treated ErbB2. A. COS-7 cells were transfected with siRNA targeting Hrs or a control siRNA, FLAG-Eps15 and PM-GFP-Ub as indicated. Proteins in equal volumes of cell lysate were separated by SDS-PAGE and analyzed by Western blotting, probing with anti-Hrs and then anti-GAPDH antibodies. C. SK-BR-3 cells transfected with siRNA targeting Hrs, or a control siRNA, FLAG-Eps15 and ErbB2-GFP and incubated with 5 μM GA for 4 hours, lysed, and subjected to SDS-PAGE and Western blotting. Equal volumes of each lysate were loaded on the gel. Blots were probed with anti-Hrs or anti-GAPDH antibodies, and then with HRP-conjugated secondary antibodies for detection by chemiluminescence. B,D. Cells transfected with the indicated constructs were processed for IF microscopy, staining with anti-FLAG and AF-594 goat anti-rabbit IgG to detect FLAG-Eps15. Merged images are shown at the right. Scale bars; 10 μm. Click here for file

Additional file 8: Figure S8 Representative Western Blot of Eps15 RNAi. A. SK-BR-3 cells transfected with siRNA targeting Eps15, or a control siRNA, were incubated with 5 μM GA for the indicated times, lysed, and subjected to SDS-PAGE and Western blotting. Equal volumes of each lysate were loaded on the gel. Blots were probed with anti-ErbB2, anti-Eps15 or anti-Hsp70 antibodies, and then with HRP-conjugated secondary antibodies for detection by chemiluminescence. B. Quantitation of bands was performed using the Odyssey infrared imaging system and the associated software. Click here for file
