# Supplementary material for: UIM domain-dependent recruitment of the endocytic adaptor protein Eps15 to ubiquitin-enriched endosomes
Source: BMC Cell Biol. 2014 Sep 27;15:34. doi: 10.1186/1471-2121-15-34 (PMC4181756; doi:10.1186/1471-2121-15-34)
Supplement: Additional file 1: Figure S1 — ErbB2 is ubiquitinated following GA treatment. SK-BR-3 cells were left untreated or incubated with 5 μM GA for 1 hour, lysed, and immunoprecipitated with anti-ErbB2. Lysates were then subjected to SDS-PAGE and Western blotting. Blots were probed with anti-ErbB2 (top panel) and anti-ubiquitin (bottom panel), and then with HRP-conjugated secondary antibodies for detection by chemiluminescence. [file 1471-2121-15-34-S1.docx]

**
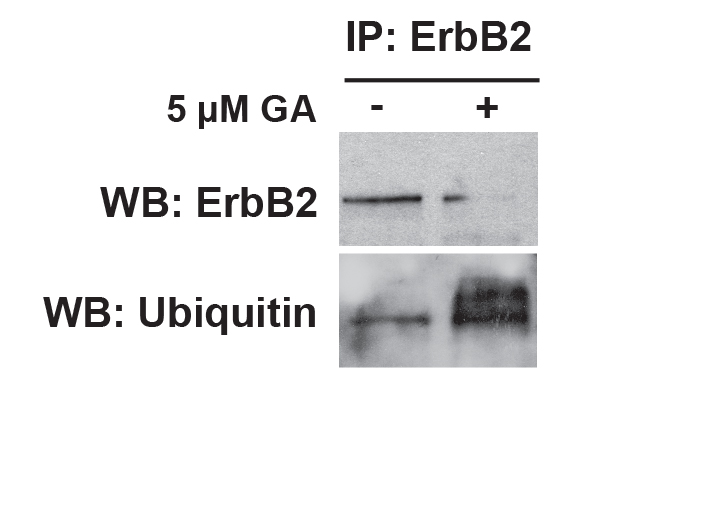
**

**Additional file 1: Figure S1.** ErbB2 is ubiquitinated following GA treatment. SK-BR-3 cells were left untreated or incubated with 5 μM GA for 1 hour, lysed, and immunoprecipitated with anti-ErbB2. Lysates were then subjected to SDS-PAGE and Western blotting. Blots were probed with anti-ErbB2 (top panel) and anti-ubiquitin (bottom panel), and then with HRP-conjugated secondary antibodies for detection by chemiluminescence.
