# Supplementary material for: UIM domain-dependent recruitment of the endocytic adaptor protein Eps15 to ubiquitin-enriched endosomes
Source: BMC Cell Biol. 2014 Sep 27;15:34. doi: 10.1186/1471-2121-15-34 (PMC4181756; doi:10.1186/1471-2121-15-34)
Supplement: Additional file 2: Figure S2 — GA does not activate Akt or MAPK. SK-BR-3 cells were either serum starved and treated with 100 ng/ml EGF for 45’ at 37°C, or treated with 5 μM GA for the indicated times, lysed, and subjected to SDS-PAGE and Western blotting. Equal volumes of each lysate were loaded on the gel. Blots were probed with anti-Akt, anti-p-Akt, anti-MAPK, anti-p-MAPK and anti-Histone H3 antibodies (loading control), and then with HRP-conjugated secondary antibodies for detection by chemiluminescence. [file 1471-2121-15-34-S2.docx]

**
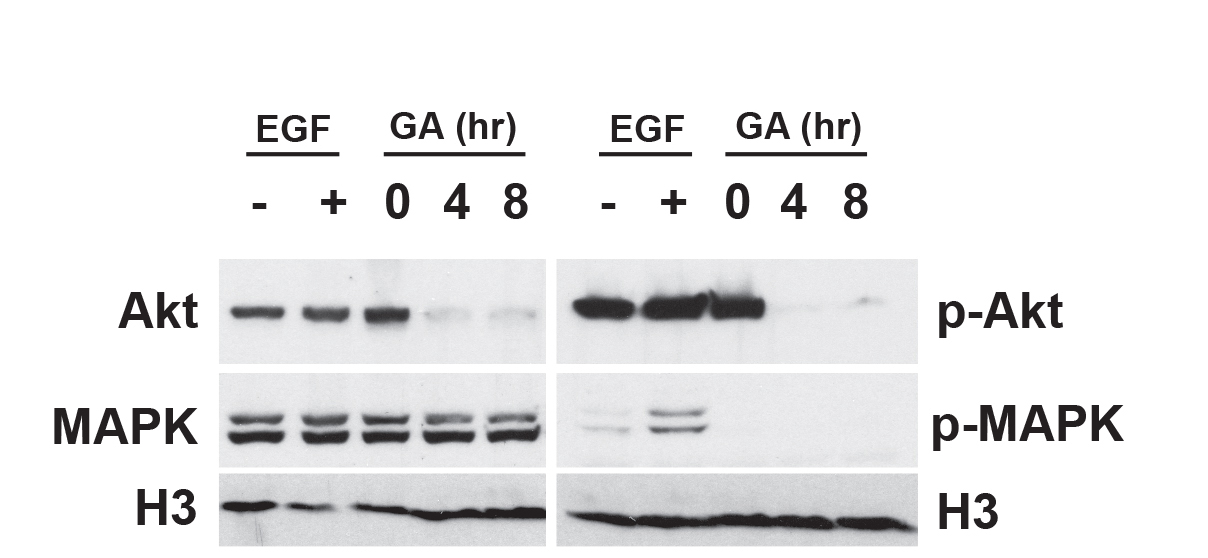
**

**Additional file 2: Figure S2.** GA does not activate Akt or MAPK. SK-BR-3 cells were either serum starved and treated with 100 ng/ml EGF for 45’ at 37**°**C, or treated with 5 μM GA for the indicated times, lysed, and subjected to SDS-PAGE and Western blotting. Equal volumes of each lysate were loaded on the gel. Blots were probed with anti-Akt, anti-p-Akt, anti-MAPK, anti-p-MAPK and anti-Histone H3 antibodies (loading control), and then with HRP-conjugated secondary antibodies for detection by chemiluminescence.
