# Supplementary material for: UIM domain-dependent recruitment of the endocytic adaptor protein Eps15 to ubiquitin-enriched endosomes
Source: BMC Cell Biol. 2014 Sep 27;15:34. doi: 10.1186/1471-2121-15-34 (PMC4181756; doi:10.1186/1471-2121-15-34)
Supplement: Additional file 3: Figure S3 — Endosomal recruitment of FLAG-Eps15 does not depend on FLAG-Eps15 expression level. FLAG-Eps15 and EEA1 were detected in FLAG-Eps15- and GFP-FYVE-UbΔGG-transfected COS-7 cells with rabbit anti-FLAG and mouse anti-EEA1 antibodies and appropriate secondary antibodies (AF-594 goat anti-rabbit IgG and AF-647 goat anti-mouse IgG). The Manders’ overlap coefficient for colocalization of Eps15 with EEA1 in each of 27 cells is plotted as function of the mean AF-594 fluorescence intensity in the same cell. [file 1471-2121-15-34-S3.docx]

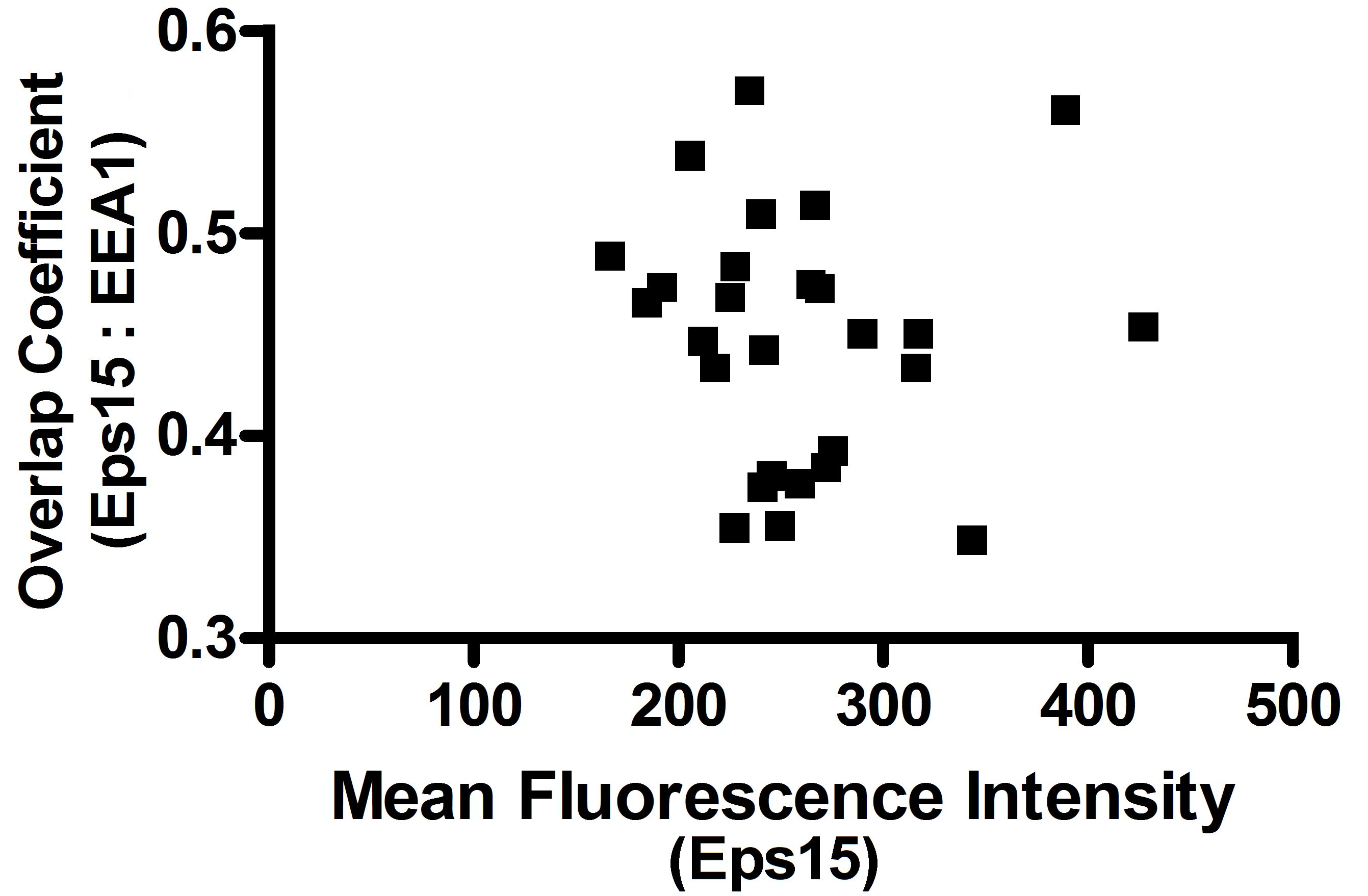


**Additional file 3: Figure S3.** Endosomal recruitment of FLAG-Eps15 does not depend on FLAG-Eps15 expression level. FLAG-Eps15 and EEA1 were detected in FLAG-Eps15- and GFP-FYVE-UbΔGG-transfected COS-7 cells with rabbit anti-FLAG and mouse anti-EEA1 antibodies and appropriate secondary antibodies (AF594 goat anti-rabbit IgG and AF647 goat anti-mouse IgG). The Manders’ overlap coefficient for colocalization of Eps15 with EEA1 in each of 27 cells is plotted as function of the mean AF594 fluorescence intensity in the same cell.
