# Supplementary material for: UIM domain-dependent recruitment of the endocytic adaptor protein Eps15 to ubiquitin-enriched endosomes
Source: BMC Cell Biol. 2014 Sep 27;15:34. doi: 10.1186/1471-2121-15-34 (PMC4181756; doi:10.1186/1471-2121-15-34)
Supplement: Additional file 4: Figure S4 — Cherry Eps15 is recruited to PM-GFP-Ub and GFP-FYVE-UbΔGG. mCherry-Eps15 was co-expressed in COS-7 cells with GFP (A), PM-GFP-Ub (B) or GFP-FYVE-UbΔGG (C), and cells were processed for IF microscopy. Scale bars; 10 μm. [file 1471-2121-15-34-S4.docx]

**
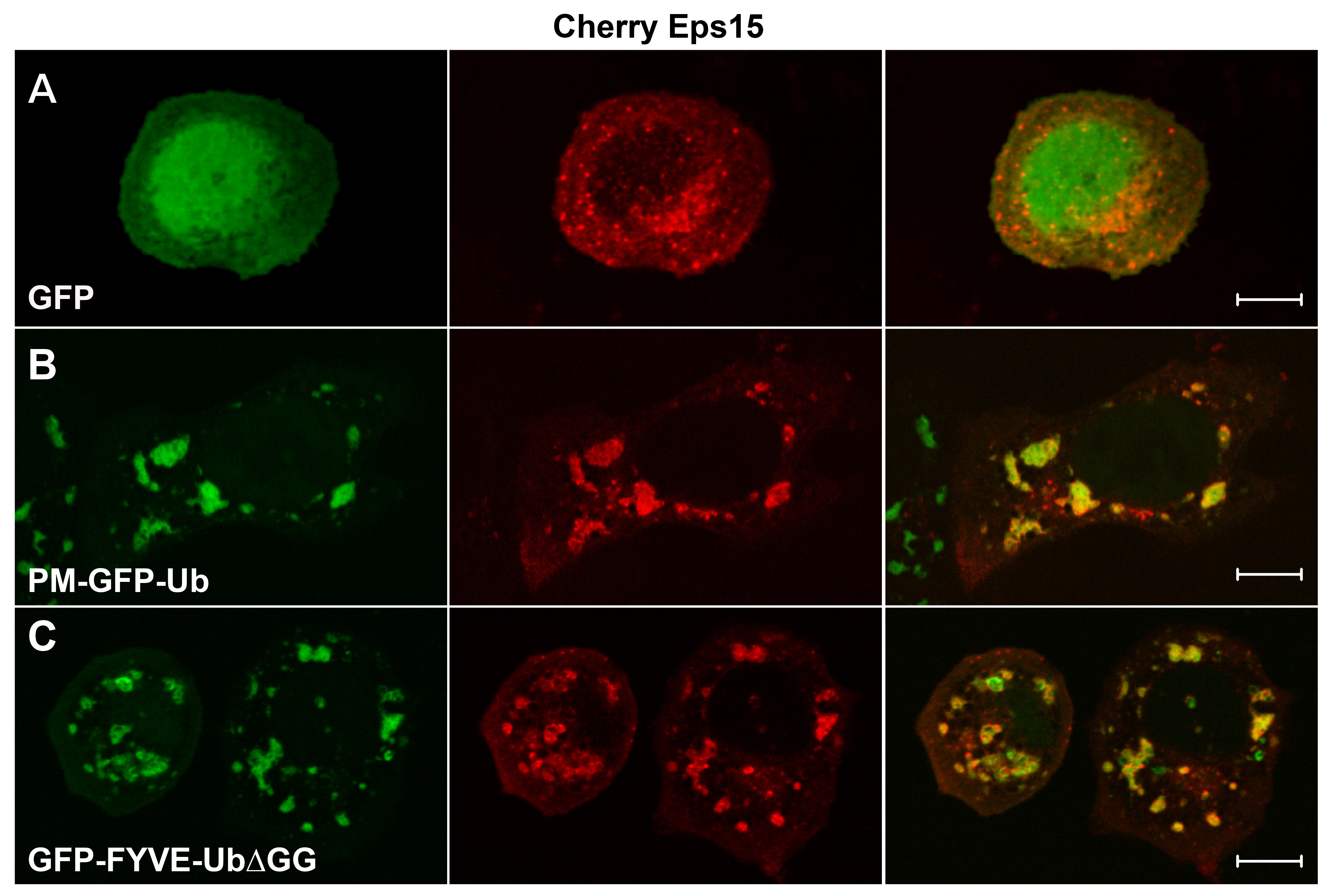
**

**Additional file 4: Figure S4.** Cherry Eps15 is recruited to PM-GFP-Ub and GFP-FYVE-UbΔGG. mCherry-Eps15 was co-expressed in COS-7 cells with GFP (A), PM-GFP-Ub (B) or GFP-FYVE-UbΔGG (C), and cells were processed for IF microscopy. Scale bars; 10 μm.
