# Supplementary material for: UIM domain-dependent recruitment of the endocytic adaptor protein Eps15 to ubiquitin-enriched endosomes
Source: BMC Cell Biol. 2014 Sep 27;15:34. doi: 10.1186/1471-2121-15-34 (PMC4181756; doi:10.1186/1471-2121-15-34)
Supplement: Additional file 5: Figure S5 — Tyr 850 is not required for endosomal recruitment of FLAG-Eps15. FLAG-Eps15 Y850F was co-expressed in COS-7 cells with either GFP (top) or GFP-FYVE-UbΔGG (bottom), and cells were processed for IF microscopy. FLAG-Eps15 Y850F was detected with anti-FLAG antibodies and AF-594 goat anti-rabbit antibodies, while EEA1 was detected with anti-EEA1 antibodies and AF-647 goat anti-mouse antibodies (pseudo-colored blue). [file 1471-2121-15-34-S5.docx]

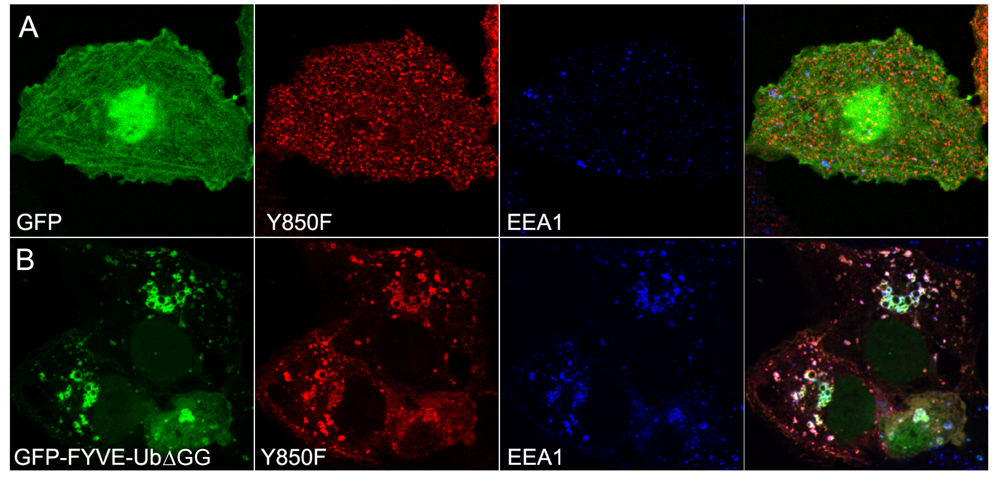


**Additional file 5: Figure S5.** Tyr 850 is not required for endosomal recruitment of FLAG-Eps15. FLAG-Eps15 Y850F was co-expressed in COS-7 cells with either GFP (top) or GFP-FYVE-UbΔGG (bottom), and cells were processed for IF microscopy. FLAG-Eps15 Y850F was detected with anti-FLAG antibodies and AF594 goat anti-rabbit antibodies, while EEA1 was detected with anti-EEA1 antibodies and AF647 goat anti-mouse antibodies (pseudo-colored blue).
