# Supplementary material for: UIM domain-dependent recruitment of the endocytic adaptor protein Eps15 to ubiquitin-enriched endosomes
Source: BMC Cell Biol. 2014 Sep 27;15:34. doi: 10.1186/1471-2121-15-34 (PMC4181756; doi:10.1186/1471-2121-15-34)
Supplement: Additional file 6: Figure S6 — Eps15 and Eps15 Y850F are recruited to activated EGFR. FLAG-Eps15 and FLAG-Eps15 Y850F were expressed in SK-BR-3 cells and were either left untreated, or stimulated with 100 ng/ml EGF for 10’ at 37°C and then processed for IF microscopy. FLAG-Eps15 and FLAG-Eps15 Y850F were detected with anti-FLAG antibodies and AF-594 goat anti-mouse antibodies, while endogenous EGFR with anti-EGFR antibodies and AF-488 goat anti-rabbit antibodies. Merged images are shown at the right with DAPI staining. Scale bars; 10 μm. [file 1471-2121-15-34-S6.docx]

**Additional file 6: Figure S6.** Eps15 and Eps15 Y850F are recruited to activated EGFR. FLAG-Eps15 and FLAG-Eps15 Y850F were expressed in SK-BR-3 cells and were either left untreated, or stimulated with 100 ng/ml EGF for 10’ at 37**°**C and then processed for IF microscopy. FLAG-Eps15 and FLAG-Eps15 Y850F were detected with anti-FLAG antibodies and AF594 goat anti-mouse antibodies, while endogenous EGFR with anti-EGFR antibodies and AF488 goat anti-rabbit antibodies. Merged images are shown at the right with DAPI staining. Scale bars; 10 μm.
