# Supplementary material for: UIM domain-dependent recruitment of the endocytic adaptor protein Eps15 to ubiquitin-enriched endosomes
Source: BMC Cell Biol. 2014 Sep 27;15:34. doi: 10.1186/1471-2121-15-34 (PMC4181756; doi:10.1186/1471-2121-15-34)
Supplement: Additional file 7: Figure S7 — Hrs is not required for recruitment of Eps15 to PM-GFP-Ub or GA-treated ErbB2. A. COS-7 cells were transfected with siRNA targeting Hrs or a control siRNA, FLAG-Eps15 and PM-GFP-Ub as indicated. Proteins in equal volumes of cell lysate were separated by SDS-PAGE and analyzed by Western blotting, probing with anti-Hrs and then anti-GAPDH antibodies. C. SK-BR-3 cells transfected with siRNA targeting Hrs, or a control siRNA, FLAG-Eps15 and ErbB2-GFP and incubated with 5 μM GA for 4 hours, lysed, and subjected to SDS-PAGE and Western blotting. Equal volumes of each lysate were loaded on the gel. Blots were probed with anti-Hrs or anti-GAPDH antibodies, and then with HRP-conjugated secondary antibodies for detection by chemiluminescence. B,D. Cells transfected with the indicated constructs were processed for IF microscopy, staining with anti-FLAG and AF-594 goat anti-rabbit IgG to detect FLAG-Eps15. Merged images are shown at the right. Scale bars; 10 μm. [file 1471-2121-15-34-S7.docx]

**Additional file 7: Figure S7.** Hrs is not required for recruitment of Eps15 to PM-GFP-Ub or GA-treated ErbB2. A. COS-7 cells were transfected with siRNA targeting Hrs or a control siRNA, FLAG-Eps15 and PM-GFP-Ub as indicated. Proteins in equal volumes of cell lysate were separated by SDS-PAGE and analyzed by Western blotting, probing with anti-Hrs and then anti-GAPDH antibodies. C. SK-BR-3 cells transfected with siRNA targeting Hrs, or a control siRNA, FLAG-Eps15 and ErbB2-GFP and incubated with 5 μM GA for 4 hours, lysed, and subjected to SDS-PAGE and Western blotting. Equal volumes of each lysate were loaded on the gel. Blots were probed with anti-Hrs or anti-GAPDH antibodies, and then with HRP-conjugated secondary antibodies for detection by chemiluminescence. B,D. Cells transfected with the indicated constructs were processed for IF microscopy, staining with anti-FLAG and AF-594 goat anti-rabbit IgG to detect FLAG-Eps15. Merged images are shown at the right. Scale bars; 10 μm.
