# Supplementary material for: UIM domain-dependent recruitment of the endocytic adaptor protein Eps15 to ubiquitin-enriched endosomes
Source: BMC Cell Biol. 2014 Sep 27;15:34. doi: 10.1186/1471-2121-15-34 (PMC4181756; doi:10.1186/1471-2121-15-34)
Supplement: Additional file 8: Figure S8 — Representative Western Blot of Eps15 RNAi. A. SK-BR-3 cells transfected with siRNA targeting Eps15, or a control siRNA, were incubated with 5 μM GA for the indicated times, lysed, and subjected to SDS-PAGE and Western blotting. Equal volumes of each lysate were loaded on the gel. Blots were probed with anti-ErbB2, anti-Eps15 or anti-Hsp70 antibodies, and then with HRP-conjugated secondary antibodies for detection by chemiluminescence. B. Quantitation of bands was performed using the Odyssey infrared imaging system and the associated software. [file 1471-2121-15-34-S8.docx]

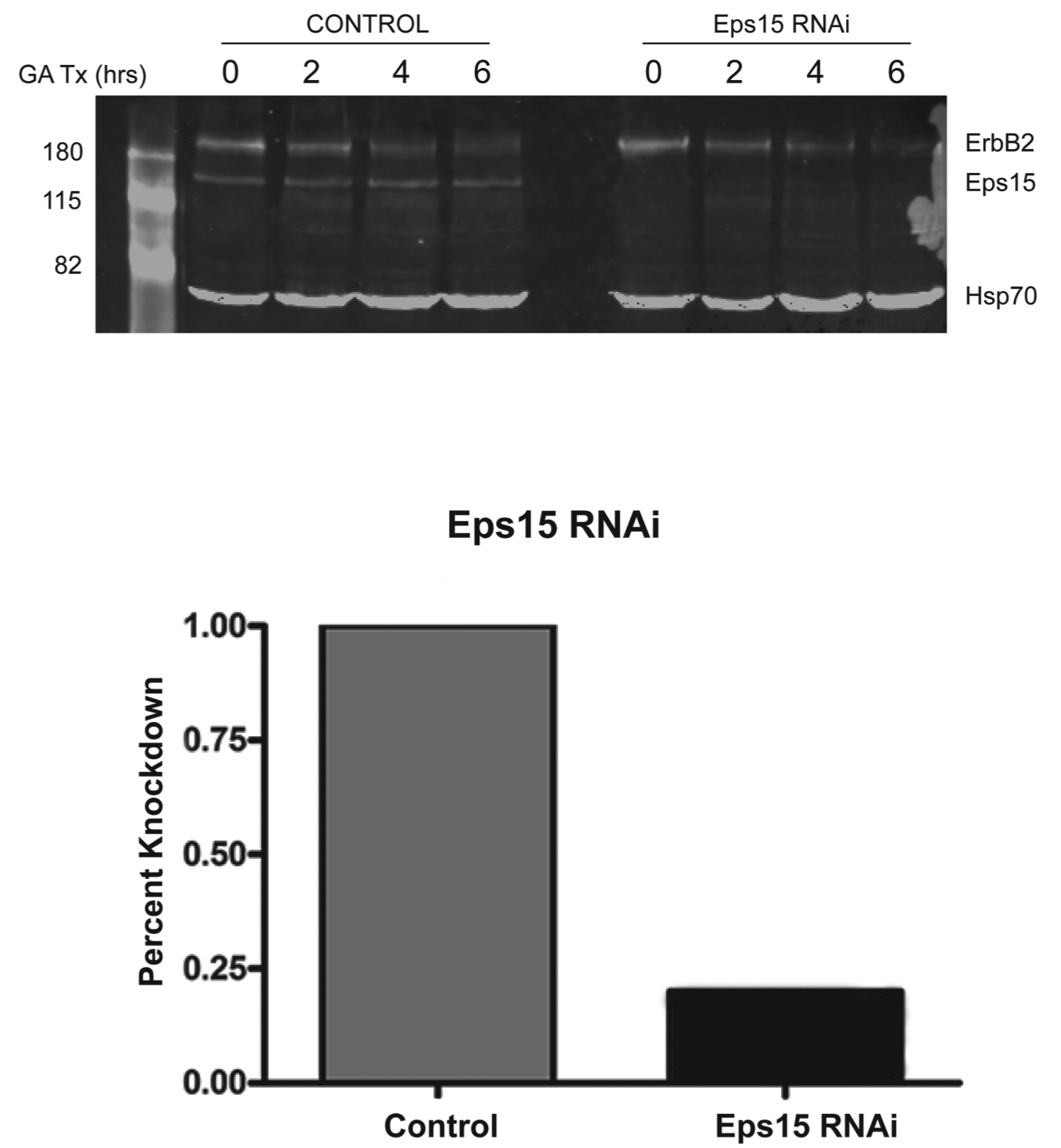


**Additional file 8: Figure S8.** Representative Western Blot of Eps15 RNAi. A. SK-BR-3 cells transfected with siRNA targeting Eps15, or a control siRNA, were incubated with 5 μM GA for the indicated times, lysed, and subjected to SDS-PAGE and Western blotting. Equal volumes of each lysate were loaded on the gel. Blots were probed with anti-ErbB2, anti-Eps15 or anti-Hsp70 antibodies, and then with HRP-conjugated secondary antibodies for detection by chemiluminescence. B. Quantitation of bands was performed using the Odyssey infrared imaging system and the associated software.
